# Preventive Measures for COVID-19 in Dental Treatments

**DOI:** 10.1017/dmp.2020.409

**Published:** 2020-10-23

**Authors:** Jincai Guo, Hui Xie, Hao Wu

**Affiliations:** Changsha Stomatological Hospital, Changsha, Hunan Province, China

**Keywords:** COVID-19, dental practice, dental treatments, preventive measures, SARS-CoV-2

## Abstract

**Objective:**

The 2019 coronavirus disease (COVID-19) has spread to most countries around the world, infecting millions of people and resulting in the pandemic. This presents great challenges for dental professionals. It is necessary to explore preventive measures for COVID-19 in dental treatments.

**Methods:**

In this paper, we outline several measures designed to prevent COVID-19 in dental treatments, such as 3-level pre-examination triage and 2-body temperature tests, 3-level protection for medical personnel, gargling before oral treatment, implementing the 4-hand operation, suctioning saliva during oral treatment, using a rubber dam, and strict enforcement of hand hygiene. These measures include recommendations for medical staff and give practical advice for managing treatment.

**Results:**

The epidemic has been brought under control, and routine oral examination and treatments have gradually been resumed from March 9, 2020, in Hunan Province, China. From January 24 to March 8, 2020, a total of 4272 patients received oral therapy during the COVID-19 epidemic in our hospital. We followed these guidelines during the COVID-19 epidemic, and none of the clinical dental staff, other workers, or patients were infected with COVID-19.

**Conclusions:**

These preventive measures for COVID-19 in dental treatments can be used as a reference for oral clinics and stomatological hospitals.

The 2019 coronavirus disease (COVID-19) is an infectious disease, which derives from severe acute respiratory syndrome coronavirus 2 (SARS-CoV-2) infection. The first case of COVID-19 was reported in Wuhan, Hubei Province, China, in December 2019. Unfortunately, the number of infections increased rapidly and quickly spread to most of the provinces in China and most countries around the world, resulting in the COVID-19 pandemic. The World Health Organization has identified it as a major global public health emergency; however, there is no specific treatment for COVID-19 at present.

COVID-19 is mainly transmitted by droplets, contact with contaminated surfaces, and aerosolized virus.^1^ According to the possible presence of SARS-CoV-2 in saliva, aerosols may play a role in SARS-CoV-2 transmission in dentistry.^2^ Dentistry presents unique challenges that may make them more susceptible to SARS-CoV-2.

Since January 2020, most provinces and cities in China have adopted a first-level emergency response to public health emergencies. The unique characteristics of dental treatments and the high risk of cross-infection prompted the suspension of all routine dental treatment services.^3^ Our hospital immediately instituted several measures to prevent COVID-19 infection, as follows.

## Methods

### Treat Only Oral Emergencies

During the outbreak of COVID-19, we adopted the general principle of treating only dental emergencies,^4^ including acute toothache, oral and maxillofacial trauma, maxillofacial space infection, temporomandibular joint dislocation, tooth trauma, spontaneous gingival bleeding, pericoronitis, and other critical patients, which were based on the health commission, dentistry-related professional societies, and quality control centers in many provinces and cities of China.

### Suspended Treatment Management

Due to the outbreak of COVID-19, medical resources were used for the prevention of COVID-19, resulting in some non-emergency diseases not being treated in a timely manner. To solve this problem, our hospital had adopted telemedicine measures. First, a 24-hour hotline was provided to patients by the specialist team. Second, video consultation was provided for patients whose problems couldn’t be solved by a telephone consultation. Third, common scientific articles and videos were pushed for advice in which urgent cases must come to the hospital and introduce self-treatment methods of each non-urgent disease for patients on the official website and WeChat.^5^

### Three-Level Pre-Examination and Triage Test and 2-Body Temperature Tests

**Figure 1:**
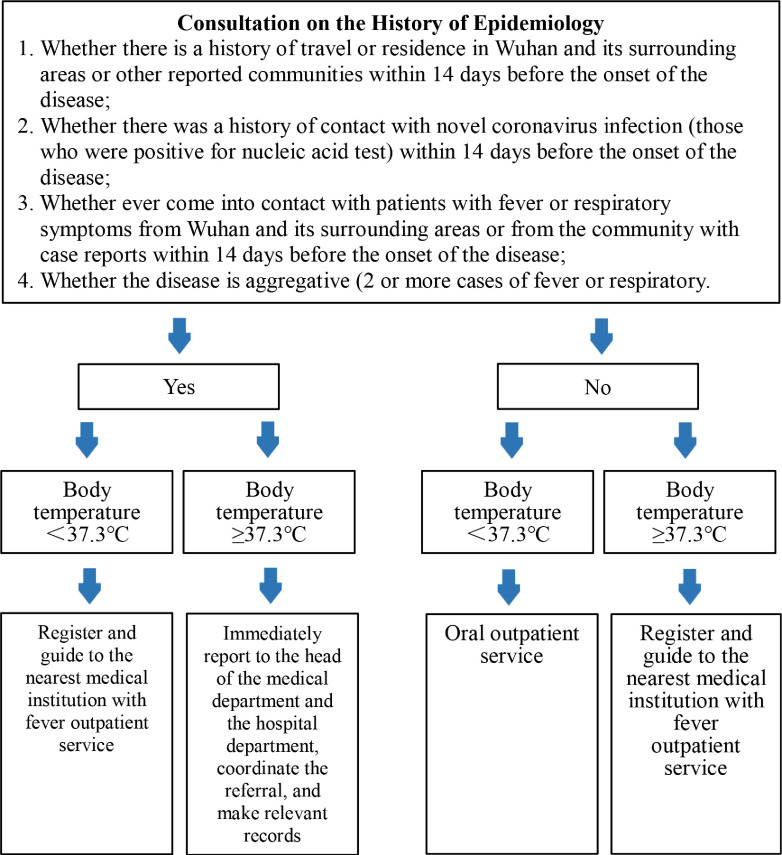
Flow Chart of Outpatient Triage.

To prevent cross-infection, the hospital instituted a 3-level pre-examination and triage test and 2-body temperature tests, patient health card status inquiry, personal itinerary query within 15 days, and prehospital screening of epidemiological history and body temperature. *Three-level pre-examination and triage* means patients must undergo 3 pre-examinations before meeting with the dentist: question and triage at the hospital entrance, more triage at the guidance desk in the outpatient hall, and before meeting the dentist in the consultation room. For the 2-temperature tests, the patient’s temperature was taken at the hospital entrance and again at the guidance desk in the outpatient hall. At the pre-examination and triage guidance desk in the outpatient hall, all patients were registered and their basic information was recorded (body temperature, ID number, telephone number, address) to facilitate future epidemiological investigations. Patients with a body temperature of 37.3°C or the epidemiological history of COVID-19 were persuaded to go to the nearest designated hospital to determine whether they were positive for COVID-19, as well as to track where they were going. Patients with suspected or diagnosed COVID-19 were sent to designated hospitals that have isolation and treatment capabilities. The pre-examination and triage flow chart is shown in Figure 1.

## Personnel Management

### Training

All staff, including medical staff, management personnel, and cleaner, received rigorous training in COVID-19 prevention via several online and on-site courses taught by infectious disease specialists. Personnel were required to master the knowledge of how to prevent COVID-19. Their knowledge was assessed by an examination after training, and anyone who failed could not be assigned to work.

### Scheduling Personnel

During the epidemic, the entire staff were evaluated daily using the WeChat app to determine whether they had any symptoms indicative of the COVID-19 infection. Only staff members who met the following criteria could be assigned to work in the dental emergency facilities: no fever, cough, fatigue, or other symptoms of potential COVID-19 infection; no history of non-local contact (outside of Changsha) within 14 days; and no history of possible exposure to the virus.

### Three-Level Protection for Medical Personnel

**Figure 2:**
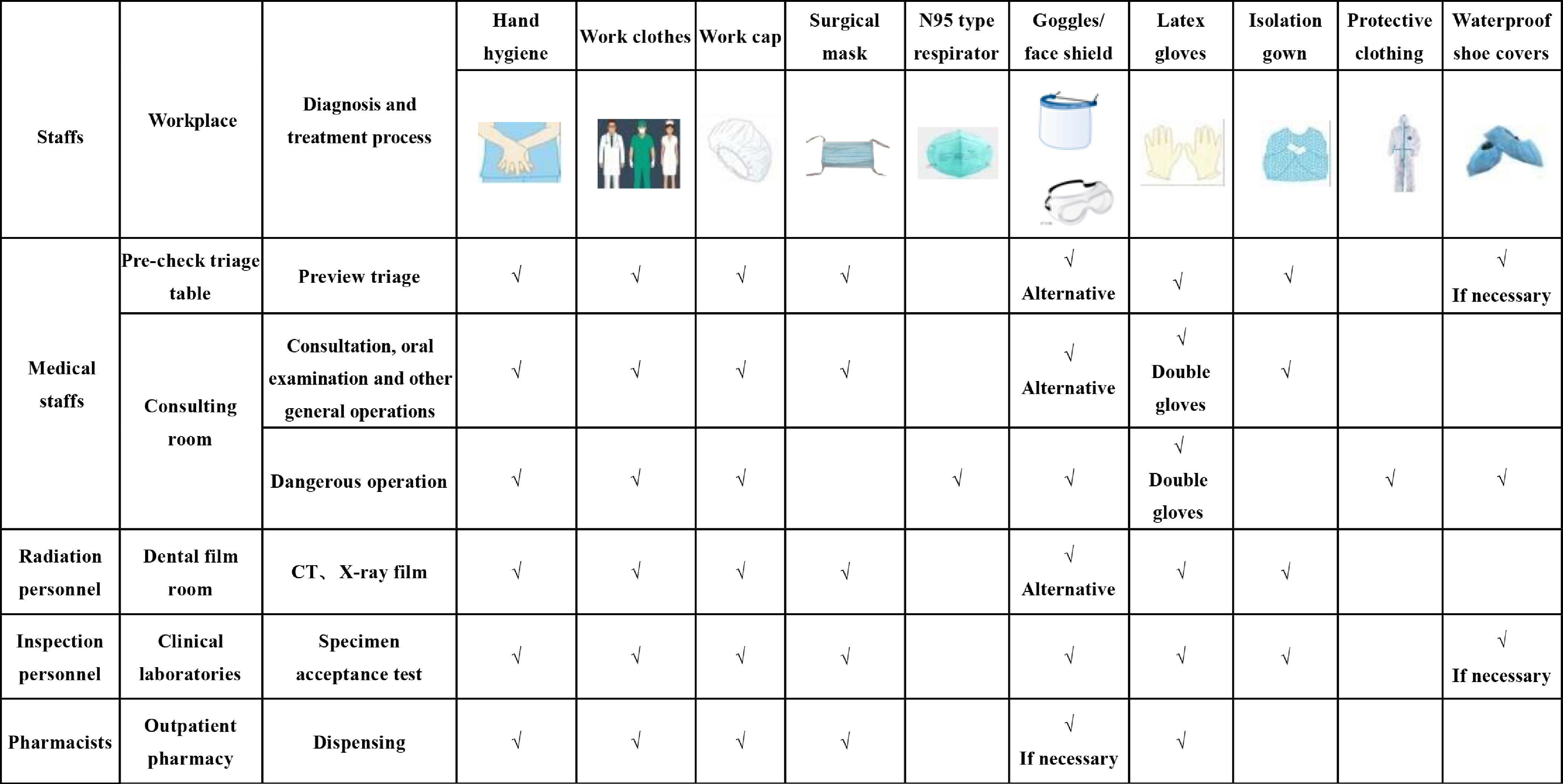
Medical Personnel Graded Protection.

The medical staff of our hospital carried out **three-level** protection. The three-level protection of medical personnel is shown in Figure 2 and described, as follows.

### Primary Protection

Oral medicine staff was required to wear disposable work caps, surgical masks, and overalls, and they could wear either protective goggles or face shields and disposable latex or nitrile gloves, if necessary.

### Secondary Protection

Disposable work caps, surgical masks, protective goggles or face shields, disposable isolation work clothes or surgical scrubs, and disposable gloves were required.

### Third-Level Protection

Disposable protective clothing, including disposable work caps, N95 type respirator, protective goggles or face shields, disposable latex gloves, and anti-leakage shoe covers, must also be worn by patients with confirmed or suspected COVID-19.

## Managing Dental Practice

### Preparing Examination and Treatment Instruments

Nurses were required to familiarize themselves with the routine and steps involved in oral examinations and treatments, to ensure that all necessary instruments and equipment were prepared in advance, and to avoid going in and out of the clinic during the examination to reduce pollution and cross-infection.

### Gargle Before Oral Examination and Treatment

Patients were instructed to gargle with 1% povidone iodine for 2 minutes before treatment, which can reduce the number of microorganisms in droplets and aerosols produced by oral procedures and surgery,^6^ and patients were shown how to seal their mouths around the mouths of a disposable water cup.

### Implement the 4-Hand Operation

During the COVID-19 epidemic, every dental chair in our hospital was equipped with a doctor and a nurse, and a 4-handed operation was carried out during oral examination and treatment. This arrangement can improve the efficiency and quality of the surgical procedure and is conducive to infection control.^7^

### Suctioning Saliva During Oral Treatment

The use of strong and weak attractors to rapidly suction saliva can also help reduce the production of droplets and aerosols during oral procedures. It is worth noting that, when using a weak attractor, patients were advised not to close their lips and bite the suction head.

### Use a Rubber Dam to Isolate the Surgical Field

The teeth are isolated using a rubber dam after induction of local anesthesia during the COVID-19 pandemic, and 75% ethanol cotton balls are used to disinfect the affected teeth and rubber dam in the surgical field. We recommend using a 3D rubber barrier without perforating because it is more convenient and quick to operate.

### Strict Enforcement of Hand Hygiene

All medical and nonmedical staff were required to strictly follow the principle of good hand hygiene during and after all examinations and treatments. The consultation room was equipped with qualified detergent, hand disinfectant, and hand-drying facilities.

## Results

On January 23, 2020, Hunan Province launched the first-level response to this major public health emergency of COVID-19, which has been downgraded to a second-level response since March 9. We described some measures taken to prevent COVID-19 transmission during the oral examinations and treatment in our hospital. From January 24, 2020, to March 8, 2020, a total of 4272 patients received oral treatment in our hospital. The dental emergency types were pulpitis (1842), periapical periodontitis (1146), pericoronitis (626), tooth trauma (420), cracked tooth (71), periodontal abscess (37), disorders of the temporomandibular joint (23), dislocation of the temporomandibular joint (21), periapical abscess (20), and others (66).

All patient basic information was registered. If a patient is confirmed with COVID-19, the information is recorded to facilitate future epidemiological investigations, which are provided to the Centers for Disease Control and Prevention (CDC). Through 3-level pre-examination and triage testing and 2-body temperature tests, we screened 50 patients with a body temperature greater than 37.3°C, 36 patients with respiratory symptoms, and 20 patients with an epidemiological history from January 24, 2020, to March 8, 2020. The CDC provides free polymerase chain reaction (PCR) tests for them. Two of them have been diagnosed with COVID-19.

We did PCR tests for all staff during the epidemic. In addition, the PCR test is also required if medical personnel have a suspected symptom or epidemiological history of COVID-19, which is evaluated daily using the WeChat app. A total of 726 PCR tests were conducted in our staff. A dentist was detected as a suspected case. The CDC quarantined her and the 16 people who came in contact with her for 14 days. Fortunately, COVID-19 was ruled out for all of them.

After implementing epidemic prevention measures, the number of outpatients in our hospital decreased from 1340 to 95 per day. After the epidemic situation was controlled, our hospital began to gradually resume routine oral examination and treatment on March 9, 2020. The number of outpatients in our hospital increased to 699 per day between March 10 and March 31 and increased to 1383 per day between April 1 and July 31. The number has since returned to the normal level. During the COVID-19 pandemic, we have followed the guidelines. All staff or patients were not infected with COVID-19. Suspected cases among them were confirmed, but, after PCR testing, results were negative.

## Discussion

We adopted the general principle of treating only dental emergencies during the outbreak of COVID-19, patients with nonacute or subacute conditions were routinely given health guidance and drug treatment, and surgery was postponed appropriately.^8^ The hospital implemented online and telephonic bookings to minimize on-site registration and to actively guide patients to see a dentist. Appointment times were staggered to reduce patient aggregation and avoid cross-infection.

It is very important to follow up the guideline of 3-level pre-examination and triage testing and 2-body temperature tests. Two COVID-19 patients were screened through the guideline, avoiding cross-infection and better protecting the safety of medical staff and patients. According to a systematic review, 88.7% of patients have symptoms of fever, which is the most obvious symptom of COVID-19.^9^ Stomatological hospitals and dental clinics can’t test for COVID-19, so checking patient temperatures and asking about the history of epidemiology are the simplest and most economical ways to screen patients.

Medical institutions have been faced with a critical shortage of protective equipment during the COVID-19 epidemic. The evidence-based medical study has found that there is no statistically significant difference in the efficacy of N95 respirators and surgical masks in the prevention of non-aerosol infection.^10^ It is suggested that N95 respirators should not be recommended for non-high-risk medical staff who are not in close contact with infected or suspected patients.

To minimize the possibility of cross-infection, it is very necessary to follow up some dental practices, such as preparing examination and treatment instruments, patient gargling before oral examination and treatment, implementing the 4-hand operation, suctioning saliva, using a rubber dam, and strictly enforcing hand hygiene.

However, the pandemic is still spreading worldwide. Therefore, all oral medicine institutions should develop and follow emergency management protocols to prevent the spread of COVID-19. Dental health workers should follow up the requirements and procedures required to prevent COVID-19.

## Conclusions

When only oral emergencies are treated during the outbreak of COVID-19, telemedicine measures, such as a 24-hour hotline, video consultation, common scientific articles, and videos, are needed. To prevent cross-infection, it is necessary for stomatological hospitals or clinics to institute 3-level pre-examination and triage testing and 2-body temperature tests. Medical staff should be required to follow strict procedures of 3-level protection. It is also necessary to strengthen the training of hospital infection for oral medical staff. Practical recommendations for managing treatment include patient gargling before oral examination and treatment, implementing the 4-hand operation, suctioning saliva, using a rubber dam, and strictly enforcing hand hygiene. These preventive measures for COVID-19 in dental treatments can be used as a reference for oral clinics and stomatological hospitals.
